# Editorial: Cognitive Diagnostic Models: Methods for Practical Applications

**DOI:** 10.3389/fpsyg.2022.895399

**Published:** 2022-04-19

**Authors:** Tao Xin, Chun Wang, Ping Chen, Yanlou Liu

**Affiliations:** ^1^Collaborative Innovation Center of Assessment for Basic Education Quality, Beijing Normal University, Beijing, China; ^2^College of Education, University of Washington, Seattle, WA, United States; ^3^China Academy of Big Data for Education, Qufu Normal University, Qufu, China

**Keywords:** cognitive diagnostic model, diagnostic feedback information, computerized adaptive test, item-level model comparison, model-data fit evaluation

Cognitive diagnostic models (CDMs) or diagnostic classification models are a relatively newer psychometric framework for collecting, analyzing, and reporting diagnostic data. They have received increasing attention in many disciplines, such as educational, psychological, and psychiatric measurement. Specifically, CDMs aim to provide discrete multivariate fine-grained diagnostic feedback information about examinees' strengths and weaknesses for developing targeted instruction and personalized support. In total, the 20 articles in this Research Topic focus on the methods and applications that contribute to the broad CDM literature by providing sustainable solutions to problems and issues emerged in practice.

Firstly, eight articles focus on providing new methods or insights that will guide the uses of CDMs in different applications for practitioners. To provide practitioners with a comprehensive guide of sample size on item recovery and classification accuracy, a thorough simulation study was conducted by Sen and Cohen to show the effects of sample size, test length, number of attributes and base rate of mastery on item parameter recovery and classification accuracy of the C-RUM, DINA, DINO, and LCDMREDUCED. Determination of the number of attributes is a fundamental issue in CDM applications. Nájera et al. evaluated the performance of a variety of dimensionality detection methods from the factor analysis literature, such as parallel analysis, minimum average partial, very simple structure, DETECT, empirical Kaiser criterion, exploratory graph analysis, and a machine learning factor forest model in discovering the number of attributes. They found that the parallel analysis with Pearson correlations and mean eigenvalue criterion, factor forest model, and model comparison with AIC are suitable in assessing the dimensionality of CDMs. A Q-matrix anchored mixture Rasch model was developed by Tseng and Wang for constructing a common scale between latent classes regardless of the ability distribution. To demonstrate the practical utility of the model, a real dataset from the Certificate of Proficiency in English was analyzed with the QAMRM, LCDM, and GDM, they find the proposed model has better model fit indices. A joint cognitive diagnostic model incorporating item responses and missing data mechanism was proposed by Shan and Wang for handling missing data not at random in the CDM framework. They demonstrate the practical value of the proposed method using the PISA 2015 computer-based mathematics data. Introducing the thinking of semi-supervised learning into CDM, Xue and Bradshaw proposed a semi-supervised learning-based diagnostic classification method using artificial neural networks. In both the simulation and real data study, they showed that the proposed method has some merit in improving the classification accuracy. The paper by Wang, Xin et al. proposed to train the BN first based on the ideal response pattern data and then to update the parameters of BN based on observed item response data using the EM or the GD algorithm. The simulation study and real data study showed the advantages of the proposed method compared to the BN training without adding the ideal response pattern data. A sequential hierarchical CDM was developed by Zhang and Wang and three absolute fit indices and five relative fit indices are used to assess model-data fit. Xu et al. proposed the slice-within-Gibbs sampler for estimating CDMs, and their simulation results confirm the viability of the sampler and show that the new method has the flexibility in incorporating a wider range of prior distributions than the Gibbs sampler.

Secondly, six articles provide use case examples of CDMs in real-world assessments, as well as demonstrate the merit and shortcoming of CDM and related model in a variety of educational and psychological research contexts. Ren et al. demonstrated that the finer-grained diagnostic feedback information from CDM can be used for targeted instruction to improve students' abilities effectively via an example with six attributes (sort, median, average, variance, weighted average, and mode) and the data distribution characteristic of the junior high school mathematics curriculum in China. Similarly, Huang et al. showed that CDMs are useful tool for developing and implementing a multi-level remedial teaching scheme, by taking electromagnetic induction as an example. Since many CDMs have been proposed, selecting the most suitable DCM for each item at the item level is a critical step in practical applications. Dong et al. compared several CDMs for the second language listening comprehension test at the test level and item level, their results verified that mixed-CDMs had a better model and person fit than the saturated GDINA model. This study provides useful insights into a better understanding of subskills and the underlying cognitive process for second language listening tests. To provide a reference for practitioners to develop parallel cognitive diagnostic tests for longitudinal learning, Tang and Zhan presented a detailed process for constructing the parallel rational number operations diagnostic tests. Three main phases of the development process are the Q-matrix and test item development, item quality monitoring, and test quality control. Liu and Bian argued that multidimensional item response theory (MIRT) model can be used for diagnostic purposes, and they compare the model-data fit of the MIRT with the reduced reparametrized unified model and generalized deterministic, noisy, and gate model for a reading comprehension test administered in China. They provide practitioners a suggestion of using the MIRT model for the diagnostic analysis for the reading test. To diagnose the strengths and weaknesses of the reading comprehension ability of the primary students, the Diagnostic Chinese Reading Comprehension Test was administered to a large population of students (*N* = 21,466) in grades 2–6 from 20 schools in a district of Changchun City, China in Li, Zhen et al. The essential components of the cognitive diagnostic assessment, attributes specification, test development, Q-Matrix validation, model comparison, reliabilities, validity, and skill profiles were reported in their study.

Thirdly, six articles focus on cognitive diagnostic computerized adaptive test and related fields. Wang, Zheng et al. proposed a new test assembly method of maximizing the minimum distance between latent classes by using mixed-integer linear programming in cognitive diagnosis, and the simulation results revealed that compared with the CDI test assembly and random test assembly, the new test assembly method had the highest accuracy rate in terms of attribute mastery pattern and attribute correct classification rates. Two item selection methods, maximum deviation global discrimination index and maximum limitation global discrimination index were developed by Li, Ma et al. to achieve better attribute coverage balance and item exposure control in the context of CD-CAT. The simulation results showed that the two new proposed methods outperformed other existing item selection methods. Similarly, Huijing et al. proposed two new item selection heuristic methods minimum parameters–information–distance method and minimum information–parameters–distance method for assembling multiple test forms, and they find that the two new methods yield better performance when the information curve of the item pool has a unimodal distribution. Tang et al. presented a simple and effective method, the theoretical construct validity to predict that the upper bound of the pattern match ratio in CDM tests through theoretical derivation and simulation study. They found that the TCV is related to the distribution of knowledge states and item categories and has nothing to do with the number of items. Wang, Tu et al. developed a hybrid optimal design for online estimation of item parameters and online calibration of the Q-matrix for new items that can be used to develop item bank effectively for cognitive diagnostic computerized adaptive testing. Three adaptive designs under different practical situations were investigated using simulation studies, the results show that the new optimal design performs better than the random design. Sun et al. investigated the influences of the calibration errors of item parameters on the variable-length cognitive diagnostic computerized adaptive testing in terms of measurement accuracy, average test length, and test efficiency. Results showed that calibration error has negative effect for the deterministic input, noisy “and” gate model and the reduced reparameterized unified model, but has less influenced for the compensatory reparameterized unified model.

In summary, this collection of papers in this Research Topic provides valuable tools, methods, insights, and examples for practitioners in obtaining diagnostic feedback information using CDMs or relevant methods.

## Author Contributions

All authors listed have made a substantial, direct, and intellectual contribution to the work and approved it for publication.

## Funding

This work was supported by the Cultural Experts, and Four Groups of Talented People Foundation of China and National Natural Science Foundation of China (grant nos. 31900794 and 32071093).

## Conflict of Interest

The authors declare that the research was conducted in the absence of any commercial or financial relationships that could be construed as a potential conflict of interest.

## Publisher's Note

All claims expressed in this article are solely those of the authors and do not necessarily represent those of their affiliated organizations, or those of the publisher, the editors and the reviewers. Any product that may be evaluated in this article, or claim that may be made by its manufacturer, is not guaranteed or endorsed by the publisher.

